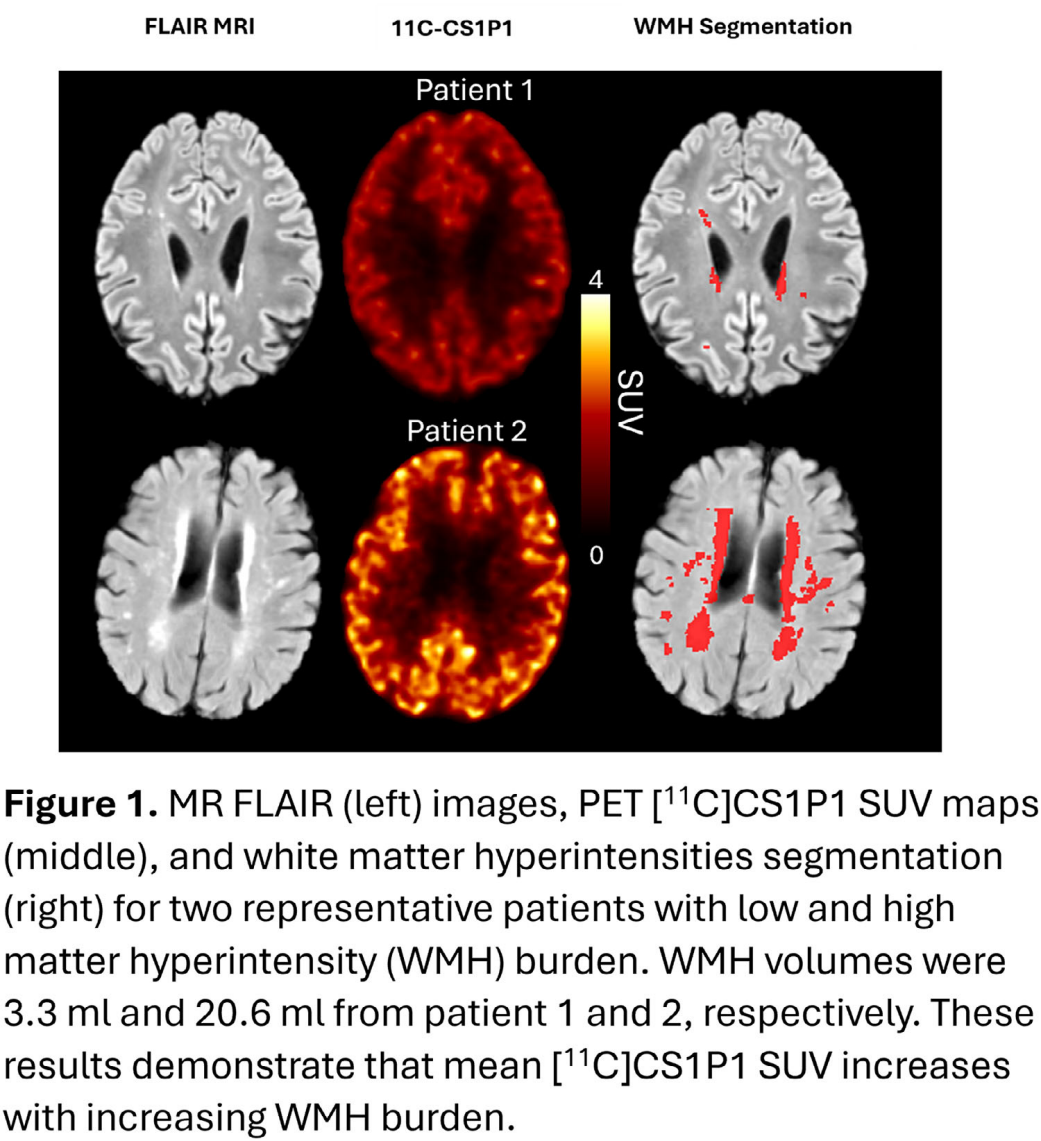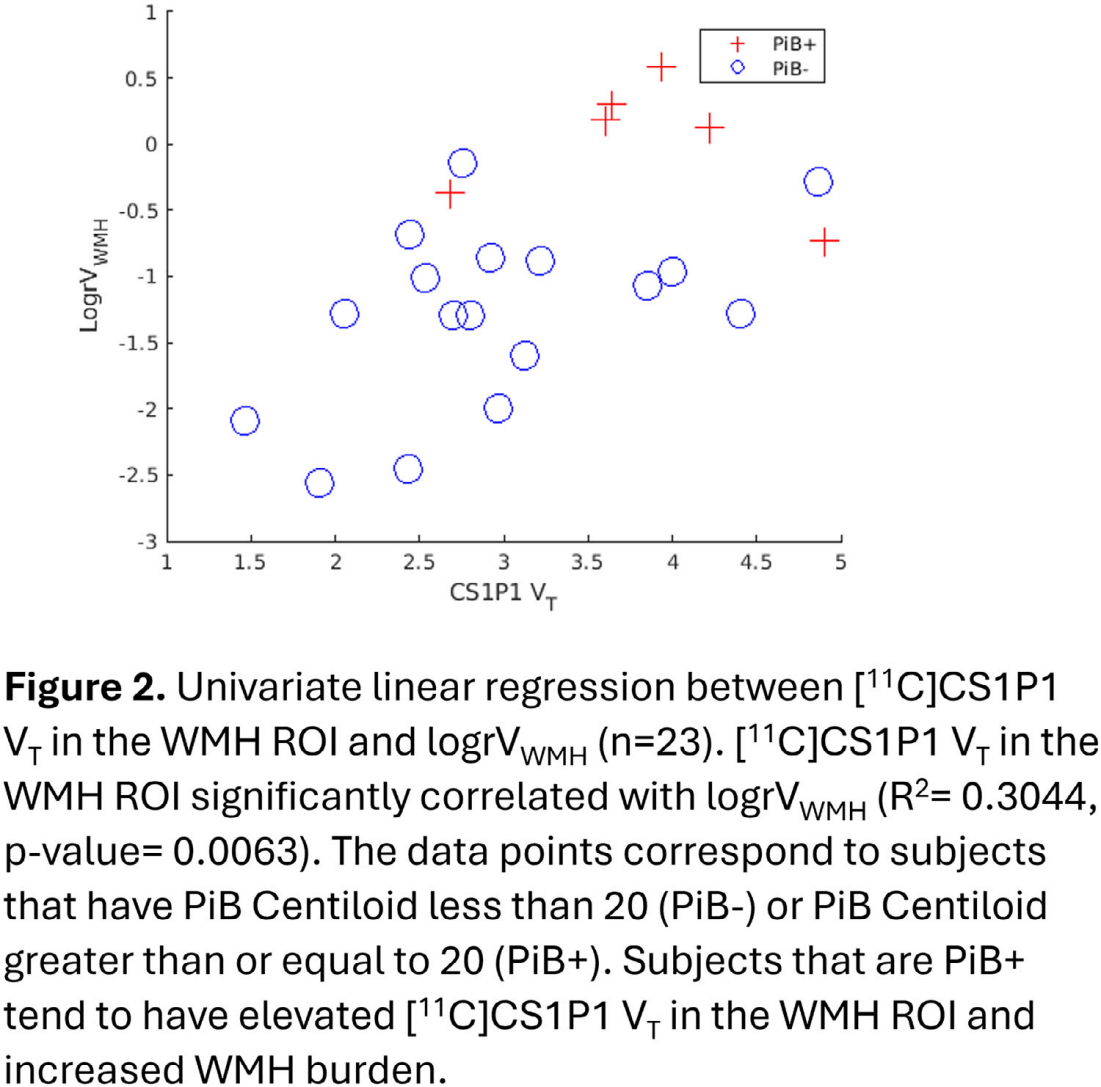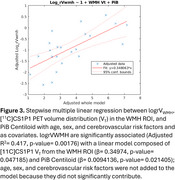# Neuroinflammation and Amyloid deposition are independently associated with white matter hyperintensity burden in AD/VCID

**DOI:** 10.1002/alz70856_105981

**Published:** 2026-01-07

**Authors:** Inema E Orukari, Yujie Wang, Yasheng Chen, Biwen Wang, Karl A. Friedrichsen, Shaney Flores, Ying Hwey Nai, Lynne A Jones, Farzaneh Rahmani, Jude‐Patrick Nnamdi Okafor, Jayashree Rajamanickam, Jin‐Moo Lee, Andria L Ford, Matthew R Brier, Zhude Tu, Tammie L.S. Benzinger, Hongyu An

**Affiliations:** ^1^ Washington University School of Medicine in St. Louis, St. Louis, MO, USA; ^2^ Washington University School of Medicine, St. Louis, MO, USA; ^3^ Washington University in St. Louis, St. Louis, MO, USA

## Abstract

**Background:**

White matter hyperintensities (WMHs) are associated with both Alzheimer's Disease (AD) and vascular contributions to cognitive impairment & dementia (VCID). Both neuroinflammation and amyloid‐β are hypothesized to contribute to WMH development, yet exact mechanisms are unknown. We have developed [^11^C]CS1P1, a novel PET probe targeting the S1PR1 receptor in the brain, a receptor which has been previously associated with neuroinflammation. In this study, we characterized [^11^C]CS1P1 PET and PiB Centiloid in human subjects with early AD/VCID.

**Method:**

We enrolled 23 subjects (≥50 years old) with varying numbers of cerebrovascular risk factors (i.e. hypertension, hyperlipidemia, diabetes, stroke, and tobacco use), and had a CDR ≤0.5. Subjects had MPRAGE MRI scans for anatomic segmentation; FLAIR MRI scans for segmentation of WMHs; [^11^C]CS1P1 dynamic PET scans for quantifying S1PR1 activity (Figure 1); and PiB scans for assessing Centiloid. A Centiloid ≥20 defined PiB positivity. MRI scans were co‐registered to [^11^C]CS1P1 PET scans, and kinetic modeling was performed for grey matter (GM), normal appearing white matter (NAWM), and WMH regions of interests (ROIs). Relative WMH volumes were normalized to whole brain volume and log‐transformed to indicate WMH burden (logrV_WMH_). Linear regression was performed to determine the relationship between logrV_WMH_ and [^11^C]CS1P1 PET volume of distribution (V_T_) in all ROIs. A stepwise multiple linear regression model was performed to evaluate the association between logrV_WMH_ and [^11^C]CS1P1 V_T_ and PiB Centiloid with age, sex, and cerebrovascular vascular risk factors as covariates.

**Result:**

[^11^C]CS1P1 V_T_ in the WMH ROI significantly correlated with logrV_WMH_ (R^2^= 0.3044, *p*‐value= 0.0063, Figure 2). Patients with high WMH [^11^C]CS1P1 V_T_ and PiB positivity had high logrV_WMH_. The stepwise multiple linear regression model demonstrated logrV_WMH_ are significantly associated with WMH [^11^C]CS1P1 V_T_ and PiB Centiloid (adjusted R^2^= 0.417, *p*‐value= 0.00176, Figure 3) after controlling for age, sex, and cerebrovascular risk factors. [^11^C]CS1P1 V_T_ in the NAWM or GM ROIs did not correlate with logrV_WMH_.

**Conclusion:**

A novel metric of neuroinflammation, [^11^C]CS1P1, and amyloid‐β are independently associated with WMH burden. Future studies are needed to evaluate whether neuroinflammation and amyloid deposition synergistically or additively contribute to WMH burden in AD/VCID.